# Hepatitis E in Italy: 5 years of national epidemiological, virological and environmental surveillance, 2012 to 2016

**DOI:** 10.2807/1560-7917.ES.2018.23.41.1700517

**Published:** 2018-10-11

**Authors:** Valeria Alfonsi, Luisa Romanò, Anna Rita Ciccaglione, Giuseppina La Rosa, Roberto Bruni, Alessandro Zanetti, Simonetta Della Libera, Marcello Iaconelli, Patrizia Bagnarelli, Maria Rosaria Capobianchi, Anna Rosa Garbuglia, Flavia Riccardo, Maria Elena Tosti, Collaborating Group

**Affiliations:** 1 Department of Infectious Diseases - Istituto Superiore di Sanità (ISS), Rome, Italy; 2 Department of Biomedical Sciences for Health - Università degli Studi di Milano, Milan, Italy; 3 Viral Hepatitis Unit, Department of Infectious Diseases - Istituto Superiore di Sanità (ISS), Rome, Italy; 4 Department of Environment and Health - Istituto Superiore di Sanità (ISS), Rome, Italy; 5 Department of Biomedical Sciences and Public Health, Virology - Hospital of Ancona, Università Politecnica delle Marche, Italy; 6 Laboratory of Virology - Istituto Nazionale per le Malattie Infettive “L. Spallanzani”, Rome, Italy; 7 National Center for Global Health - Istituto Superiore di Sanità (ISS), Rome, Italy; 8 Collaborating Group members have been listed at the end of this article

**Keywords:** Hepatitis E, Italy, surveillance, risk factors, viral infections, epidemiology

## Abstract

Increasing numbers of hepatitis E cases are being reported in several European countries, including Italy, but the burden of hepatitis E virus (HEV) infection is largely unknown in the latter. To gain a better understanding of HEV epidemiology at national level in Italy, we piloted a strengthened and integrated human (epidemiological and virological) and environmental HEV surveillance system between 2012 and 2016. Over the 5-year period, 169 confirmed hepatitis E cases were identified, with a national annual incidence of 0.72 cases per 1,000,000. Of 65 HEV-RNA positive samples of sufficient quality for molecular analysis, 66% were genotype HEV3, 32% HEV1 and 1% HEV4. The most frequent risk factor reported by all HEV3 infected cases, was the consumption of undercooked pork and sausage. For the environmental surveillance, 679 urban sewage samples were collected from 53 wastewater treatment plants and HEV-RNA was detected in 38/679 of the samples. Among these, 25 (66%) were genotype HEV3 and the remaining were HEV1. We demonstrate that autochthonous transmission and environmental circulation of genotype HEV3 is adding to travel-related HEV transmission in Italy. We recommend the ‘One Health’ approach to integrated surveillance, and to include HEV-related messages within health information campaigns focussing on food security.

## Introduction

Hepatitis E is a systemic disease predominantly affecting the liver and caused by infection with the hepatitis E virus (HEV). Although the burden of hepatitis E worldwide is unknown, it is estimated that one-third of the world population has been exposed to the virus at some time during their lives [1]. Each year, 20 million people are estimated to acquire HEV infections, with over 3 million developing symptomatic disease and almost 60,000 HEV-related deaths [2].

The global distribution of HEV has distinct epidemiological patterns based on ecology and socioeconomic factors. In resource-poor countries, hepatitis E disease presents as large-scale waterborne epidemics of which a few have spread through person-to-person contact [2]. Conversely in the Western world, hepatitis E was traditionally considered a travel related disease, even though the perception has changed due to the increasing number of autochthonous cases reported in European Countries in recent years; at present it is recognised that HEV is endemic in the European Union/European Economic Area (EU/EEA) [3]. Of the seven HEV genotypes known to infect humans (HEV1–4 and HEV7) and animals (HEV3–6), EU/EEA countries report mainly HEV3 autochthonous infections [4].

In Italy, viral hepatitis is a notifiable disease. However, the statutory surveillance system does not specifically collect data on HEV but more generically on viral hepatitis. Since 2007, to support the statutory system, the notification of acute HEV infection was included in the existing parallel enhanced surveillance system for viral hepatitis called SEIEVA (Integrated Epidemiological System for Acute Viral Hepatitis) [5] managed by the Italian Institute of Health (ISS). In parallel, a HEV environmental surveillance, also managed by ISS, analyses urban sewage samples collected in the framework of a wastewater treatment plant (WTP)-based network throughout Italy [6].

Between 2012–2016, motivated by the evidence of a Europe-wide increase in HEV reported cases [4] and by the lack of reliable data on HEV at national level, a strengthened human (epidemiological and virological) surveillance of acute symptomatic HEV cases and a tentative integration with environmental HEV surveillance, was implemented to gain a better understanding of current HEV epidemiology in Italy. Here, we show results of this pilot surveillance system, with the perspective of establishing a well-functioning and integrated HEV surveillance system.

## Methods

### Case definition and data collection

The present study was performed within the SEIEVA surveillance [5]. One hundred and fifty-one of 189 Italian Local Health Units (LHU) voluntarily participate to the surveillance and collect demographic and epidemiological information for all acute viral hepatitis, meeting the criteria included in the World Health Organization (WHO) case definition [7]. In addition from 2012 the participating LHU were required to enhance reporting of HEV cases and to collect more specific information on HEV risk factors (occupational and food-related risks) in the 6 weeks before symptom onset, through an additional data sheet. If HEV biomarkers were not available, LHU were required to report any acute viral hepatitis cases negative for hepatitis A, B and C viruses (defined as possible cases for the purpose of this study).

### Human samples

The sera from the cases were sent to one of four reference laboratories for confirmation, including the National Reference Laboratory for Viral Hepatitis (ISS, Rome) or one of three Regional Reference Laboratories i.e. Lombardia (Università di Milano, Milan), Marche (Università Politecnica delle Marche, Ancona) and Lazio (Istituto Nazionale per le Malattie Infettive “L. Spallanzani”, Rome).

Amplicon sequencing of all positive samples was also performed by the reference laboratories. HEV RNA was extracted and amplified by nested reverse transcription polymerase chain reaction (RT-PCR) using two sets of primers targeting ORF1 (172 bp) and ORF2 (348 bp) using a consolidated methodology [8]. Purified PCR amplicons were then subjected to bidirectional automated sequencing. We assembled the raw forward and reverse ABI files into a single consensus sequence using MEGA 6.06 software. We submitted all sequences to Basic Local Alignment Search Tool (BLAST) analysis for genotyping.

### Environmental samples

Urban sewage samples were collected from 53 wastewater treatment plants from 16 of the 21 Regions in Italy. Wastewater samples were handled and analysed as previously described [6] and amplification and sequencing were performed, in the same way as for clinical samples, by extracting viral nucleic acids that were from chloroform-treated samples of wastewater and amplifying them using nested RT-PCR.

### Analytical study and statistical analysis

A descriptive analysis of the data was conducted; continuous variables were presented as medians and ranges and categorical variables as percentages. The total percentages of reported risk factors can exceed 100% since several mentions were possible. The incidence of reported hepatitis E cases was computed using, as a denominator, the population residing in all the LHUs reporting to SEIEVA. The 95% confidence intervals (95% CI) of incidence rates were calculated assuming a Poisson distribution of observed cases.

In addition, the confirmed hepatitis E cases were compared with nonA-nonE Hepatitis cases using non-parametric Mann–Whitney test, for continuous variables, and chi-squared test (or Fisher’s exact test, when necessary) for categorical variables.

In addition, a case–control study was conducted to estimate the strength of the association between HEV infection and different risk factors, using nonA-nonE cases as the control group. In order to improve power, eight additional confirmed hepatitis E cases that arose during the first months of 2017 were added to the dataset. The association between HEV infection and the considered risk factor was estimated by odds ratios (ORs) and their 95% CI, adjusted using the multivariate logistic regression model; factors significantly associated in univariate analysis were included in the model. P values < 0.05 were considered as statistically significant.

All data were analysed by Stata Statistical Software, version 13.1, (StataCorp, Lakeway Drive College Station, Texas, United States).

## Results

From 1 January 2012 to 31 December 2016, 456 of 5,057 (9.0%) cases notified to the SEIEVA were possible hepatitis E cases. Of those, 293 (64.3%) were tested for IgM anti-HEV. Tested cases were more frequently from, or had travelled to, highly endemic areas. No other statistically significant differences in socio-demographic characteristics were observed between tested and non-tested possible hepatitis E cases. A total of 169 (57.7%) confirmed hepatitis E cases and 124 (42.3%) nonA-nonE hepatitis cases were identified. Therefore, during the study period, 169 (3.3%) of all hepatitis cases notified to SEIEVA (5,057) were definitely ascribed to HEV. The Figure shows monthly distribution of confirmed hepatitis E cases. A mean of 2.8 cases per month, with no time trend, was observed.

**Figure:**
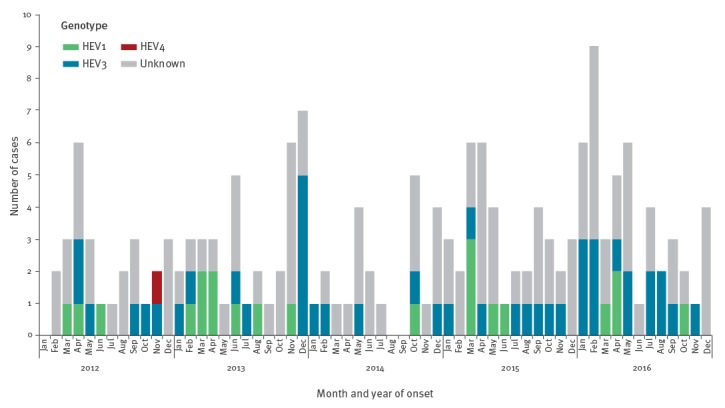
Distribution of confirmed hepatitis E cases notified by month and year of notification, Italy, 2012–2016 (n = 169)

HEV: hepatitis E virus.

Since only 293 (64.3%) of 456 possible hepatitis E cases were tested, we calculated the national incidence rate of confirmed Hepatitis E cases to be at least 0.72 cases per 1,000,000 inhabitants per year (95% CI: 0.62–0.84). Incidence rates ranged from 0.96 per 1,000,000 (95% CI: 0.71–1.29) in 2016 to 0.47 per 1,000,000 (95% CI: 0.29–0.71) in 2014. At a regional level, a significant difference in incidence was observed only in the Marche region (Central Italy) with 4.08 per 1,000,000 confirmed hepatitis E cases per year (95% CI: 2.75–5.82) but 6.12 (95% CI: 2.80–11.6) in 2013. The increased incidence in 2013, could be explained by a concurrent outbreak involving eight cases between December 2013 and February 2014; the median age of these outbreak cases was 61 years (range 41–67) and all but one were male. Seven cases reported sausage consumption (information not available for one case), all were infected by genotype HEV3.

From 2012–2016, serum samples were collected from 91/169 (53.8%) confirmed hepatitis E cases for HEV-RNA determination of which, 73 (80.2%) were positive for HEV-RNA and 18 HEV-RNA were not detectable. Of the 73 HEV-RNA positive samples, 65 were adequate for the molecular analysis. Of those, 43 (66.2%) were genotype HEV3, 21 (32.3%) HEV1 and one (1.5%) HEV4 (Figure). Genotype distribution was associated with age, with HEV3 infected cases being older than those infected by HEV1 (median age 58 and 31 years, respectively; p < 0.001). No significant difference in the clinical presentation and laboratory parameters was observed. All non-Italian cases except for one (n = 18) were infected by HEV1, the other by HEV4. Conversely, almost all Italian cases (37/39; 94.9%) were infected by HEV3, the remaining two cases were infected by HEV1.

With the inclusion of the additional eight confirmed hepatitis E cases that occurred in 2017, the analytical study of risk factors was performed on 177 confirmed hepatitis E cases. Of those, 139 were males (78.5%). The male to female ratio was 3.7:1. The median age was 50 years (range 16–87 years); no cases were reported among children. One hundred and fourteen cases (68.7%) were Italian, 52 non-Italian nationals 15 (28.9%) were from Bangladesh, 11 (21.2%) from Pakistan and 12 (23.1%) from India. The majority of notified cases were diagnosed in Northern and Central Italy (Table 1).

**Table 1:** Comparison between hepatitis E and nonA-nonE cases on demographic characteristics, Italy, 2012–2016 (n = 301)

Demographic Characteristics	Hepatitis				p-value
	E (n = 177)		nonA-nonE (n = 124)		
	n	%	n	%	
**Age group (years)**					
10–30	33/175	18.9	19/120	15.8	0.180
31–45	39/175	22.3	40/120	33.3	
46–60	58/175	33.1	31/120	25.8	
61–87	45/175	25.7	31/120	25.0	
**Sex**					
Male	139/177	78.5	75/123	61.0	0.001
Female	38/177	21.5	48/123	39.0	
**Nationality**					
Italian	114/166	68.7	103/122	84.4	0.002
Non-Italian	52/166	31.3	1/1229	15.6	
**Geographical area of diagnosis**					
North	72/170	42.4	56/124	45.2	0.155
Centre	94/170	55.3	60/124	48.4	
South/Islands	4/170	2.3	8/124	6.4	

Different denominators for age group, sex, nationality and geographical area of diagnosis were due to missing information.

Of the confirmed hepatitis E cases with available information, 74% (101/137) presented with jaundice, 22/51 (43%) with fever, 20/51 (39%) had an unexplained weight loss and 19/52 (37%) abdominal pain. No neurological disorder was reported.

Almost all of the confirmed cases with available information 150/160 (93.8%) required hospitalisation (median length 10 days; range: 2–51). Four cases had complications: fulminant hepatitis and two deaths, of which blood samples were not available. One case of fulminant hepatitis occurred in 2012 in a woman in her late-20s from Pakistan who subsequently underwent liver transplantation. The second case was observed in 2013 in a man in his mid-60s from Italy, who recovered spontaneously. The two deaths occurred in 2014 in two Italian men in their 80s. Both had underlying liver cirrhosis (one HBV-related). Two HEV chronic cases, both infected with HEV3, were reported in 2012, one in a man in his early 60s from Italy with underlying malignant disease and haemolytic anaemia [9] and the other case was in an immunosuppressed Italian woman in her mid-60s [10].

### Analytical study

Confirmed hepatitis E cases were more frequently male compared with nonA-nonE cases (78.5% vs 61.0%, respectively; p = 0.001) and of non-Italian nationality (31.3% vs 15.6%, respectively; p = 0.001) (Table 1). No significant difference in the clinical presentation and laboratory parameters was observed.

The most frequently reported risk factor among confirmed hepatitis E cases was the consumption of undercooked pork (45/57; 79%), and undercooked pork sausages (32/52; 62%). All HEV3 infected cases, with available information, reported one or more of these risk factors. Of the 135 confirmed cases with available information, 64 (47%) reported shellfish consumption (38% ate raw shellfish), and all but three were infected by HEV3.

Of the 163 confirmed hepatitis E cases with available information, 42 (26%) reported travelling to South-Asia during the 6 weeks before symptom onset (mainly India, Bangladesh and Pakistan); this exposure was more frequent among non-Italian nationals (n=34/49; 69%). All these cases were infected with genotype HEV1 (one Italian case with a travel history to India and 16 non-Italian cases travelling to their country of origin in South Asia). The HEV4 infected case was Croatian with a travel history to Ukraine. Blood transfusion was reported in one case (genotype information was not available).

Confirmed hepatitis E cases were significantly more often exposed to eating undercooked pork/sausage than nonA-nonE cases. Multivariate analysis showed a more than fourfold increased risk of hepatitis E associated with pork consumption and a threefold risk associated with sausages consumption (Table 2).

**Table 2:** Comparison between hepatitis E and nonA-nonE cases by risk factors, Italy, 2012–2016 (n = 301)

Risk factors	Hepatitis				p-value (univ.)	OR_adj_^a^ (95% CI)
	E (n = 177)		nonA-nonE (n = 124)			
	n	%	n	%		
Travel	61/163	37.4	32/116	27.6	0.086	NA
Shellfish consumption	64/135	47.4	35/82	42.7	0.498	NA
Raw (among shellfish consumers)	21/55	38.2	6/16	37.5	0.961	NA
Berries consumption	13/94	13.8	5/22	22.7	0.299	NA
Drinking well water	15/130	11.5	3/45	6.7	0.354	NA
Pork consumption	45/57	79.0	30/61	49.2	0.001	4.6 (1.3–16.1)
Undercooked sausages consumption	32/52	61.5	16/48	33.3	0.005	2.9 (1.1–7.6)

CI: confidence interval; NA: not applicable because not inserted in the model; OR_adj_: adjusted odds ratio.

^a^Adjusted for age, sex, nationality and the respective other listed factor.

Among the sub-group of cases with no travel history to endemic areas (116), the association with pork consumption was 5.7 (95% CI 1.5–20.8).

HEV-RNA was detected in 38 of 679 (5.6%) raw sewage samples, of these, 25 were characterised as HEV3 and 13 were HEV1. HEV was detected in nine of 16 Regions throughout Italy: 4.5% (13/290) of samples were positive in Northern Regions; 7.2% (8/111 samples) in Central Regions and 6.1% (17/278) in Southern Regions, the difference was not statistically significant (p = 0.505).

## Discussion

HEV infection is more widely distributed in Europe than previously thought and is spread across endemic and non-endemic areas by travel and zoonosis [3]. An increasing number of HEV cases are no longer occurring exclusively among returning travellers from endemic areas. Such cases are being reported more often in several European countries e.g. France [11], the UK [12] and Germany [13]. It is the converse in Switzerland, notwithstanding a steady increase in demand for molecular testing, the proportion of positive HEV samples has remained roughly constant over the years from 2011 to 2016 [14]. HEV circulation in Italy was demonstrated since the 1980s and sporadic autochthonous incident cases have been reported for several years [15]. Despite this, the real burden of HEV-related diseases is largely unknown in Italy.

We found that the estimated acute hepatitis E incidence was very low in Italy, with more than 60% of confirmed cases having genotype HEV3. We observed no time trends over the study period (2012–2016) but did find notable difference in HEV incidence in Marche Region (Central Italy). This may indicate that HEV only provides a small contribution to the overall acute viral hepatitis burden in Italy, which is mainly sustained primarily by hepatitis A virus (HAV) and hepatitis B virus (HBV) (48.4% and 32.2%, respectively) [SEIEVA data not shown]. However, the low incidence of HEV might also be a result of an underestimation of the true morbidity, which could be due to three main reasons.

First, underestimation of HEV incidence could be the result of an unknown number of un-notified asymptomatic/sub-clinical HEV infections. This hypothesis is supported by the fact that previous seroprevalence studies conducted in Italy showed relatively high prevalence rates of anti-HEV IgG antibodies ranging between 1–10% [16,17] and more recently, a very high seroprevalence (49%) was observed among blood donors from the Abruzzo region [18]. Despite the considerable heterogeneity found between studies, which was mainly attributable to the assay employed and the geographical location, the HEV seroprevalences may suggest a wider circulation of HEV than clinically observed. This heterogeneity observed in different parts of Italy is consistent with other countries e.g. France, where HEV is highly endemic only in southern and north-eastern areas of the country [19]. This heterogeneity was also found in hepatitis E seroprevalence rates across Europe that range from 0.6% to 52.5% [20].

Second, underestimation of hepatitis E incidence could be the result of case under-ascertainment, with only one third of possible hepatitis E cases being tested for HEV infection during the study period. This underestimation seems to mainly affect cases with genotype HEV3, as possible hepatitis E cases tested were from, or had travelled to, HEV1-endemic areas more frequently than cases who were not tested. A non-homogeneous ascertainment of disease could, therefore, have contributed to the observed geographical distribution of hepatitis E cases, since a different awareness of HEV infection among clinicians or health services could have led to higher or lower probabilities of samples being tested for HEV. Further, under-ascertainment could affect disproportionally regions with little or no diagnostic capacity for HEV. This finding is supported by the results from environmental surveillance that conversely showed no geographic difference in HEV distribution across Italy. A survey conducted in 2016 (unpublished data) revealed that laboratory diagnostic capacity for HEV infection is absent in almost half of all Italian regions. When present, different HEV IgM assays were used. This diagnostic capacity was probably poorer in 2009 [21].

Third, the lack of standardised serological diagnostic tests and variant performance of IgM and IgG HEV assays may impact the diagnosis. This should be taken into account when considering the establishment of a regular national surveillance of acute infection, as it could lead to an underestimation of hepatitis E incidence due to an unknown number of false negatives. However, in our study we classified confirmed hepatitis E cases by serological positivity, according to the WHO case definition [7]. Nevertheless, anti-HEV IgM ELISA was demonstrated to be a good screening test for surveillance purposes and not limited by inconsistent performances of sensitivity and specificity among different assays [22].

Here, we found that two main risk factors for acquiring hepatitis E in Italy were linked to specific viral strains. Cases with genotype HEV1 were associated with travel to highly endemic areas. Whereas, non-travel related cases were associated with infection with genotype HEV3, which was locally acquired. The HEV3 cases were significantly older than HEV1 cases and more than 70% of cases reported consumption of undercooked pork/sausage, which is in line with the fact that HEV3 can be transmitted via the consumption of raw or undercooked meat of infected animal reservoirs (e.g. domestic pig, wild boar and deer) [23]. The multivariate analysis showed a fourfold greater risk of HEV3 infection after the consumption of undercooked pork, particularly an Italian sausage usually consumed raw. This association between HEV3 and undercooked pork was stronger when travel related cases were excluded from the analysis, thus supporting that autochthonous HEV circulation in Italy is primarily related to food habits.

In Italy, pork is the most frequently consumed meat product with an estimated *pro capita* yearly consumption of 36.2 kg in 2015 [24]. Several Italian regions have traditional dishes based on raw/undercooked pork products (sausage in particular) and home-based processing of pork is still common in rural areas. This peculiar food habit could contribute to regional differences in HEV endemicity; a speculation supported by the outbreak that occurred in the Marche region linked to the consumption of a locally produced raw pork liver sausage. In addition, seroprevalence of HEV in domestic pigs is relatively high in Italy, indicating an active circulation of the virus. A recent study found a 50% seroprevalence in swine from 42 farms located in north-western Italy [25]; all HEV strains isolated from wild boars and domestic pigs in Italy are HEV3 [25,26].

HEV transmission has been also described via blood and blood component transfusions. For this reason, donor screening for HEV-RNA is under serious consideration in some developed countries [27,28]. In our surveillance, blood transfusion was a reported risk factor in only one case, suggesting that transmission of HEV through this route might be limited in Italy.

From a clinical standpoint, findings from our study were in line with the usually described presentation of HEV3 infection, which is not associated with high mortality rates, like HEV1, but can cause chronic infection in immunocompromised or immunosuppressed patients [23,29]. We documented two fatal cases (both in patients with underlying chronic liver disease) and two immunocompromised cases (in which HEV infection became chronic) [9,10] but most cases presented with a mild and self-limiting disease.

A low frequency of HEV-RNA positive samples were found in urban wastewaters, possibly due to the low incidence and prevalence of HEV3 strain in humans. One third of all positive environmental samples were characterised as HEV1, indicating that HEV1 is also present in Italy; confirmed by the occurrence of human cases associated to travel to highly endemic areas. In contrast, non-travel related cases were all infected with HEV3, locally acquired, suggesting that HEV1 is not endemic in our country. In terms of environmental spread, HEV was detected in urban wastewaters collected from nine of 16 Italian regions, where clinical cases occurred in all but two (one region was not covered by the SEIEVA surveillance). The occurrence of HEV in urban wastewater raises the question whether sewage-contaminated surface waters can also contribute to HEV endemicity in our country. Recently, HEV has been detected from Italian river waters that are receiving wastewater discharges [30]. Moreover, HEV strains have been isolated from shellfish, marine waters and underwater sewage discharges in Italy [31], suggesting the possibility of HEV transmission via the waterborne route.

### Strengths and limitations

The study was an enhanced HEV surveillance set in the framework of a national surveillance system (SEIEVA) that covers 80% of the Italian population distributed throughout the country. It was performed through the merging and integration of different data sources of HEV information in Italy, providing a representative overview of the HEV situation across Italy. It is possible that the incidence of HEV was underestimated, however, as testing capacity/habits resulted in just 64% of possible cases tested.

Another possible limitation is due to the widespread consumption of pork in Italy, making the direct association of hepatitis E with pork consumption difficult to estimate precisely. To limit this possible bias, we used a control group (nonA-nonE) that was selected from the same population as the HEV affected group and thus were assumed to have access to the same food products.

To our knowledge, this is the first study to provide a comprehensive overview of HEV epidemiology in Italy, following the European Centre for Disease Prevention and Control (ECDC) recommendation for a common EU strategy to better understand case numbers and determine circulating strains of this virus [3]. The positive experiences during the pilot led to a consolidation and improvement of an integrated surveillance approach for HEV.

## Conclusion

The observed low incidence of hepatitis E in Italy could be due to under-notification and under-ascertainment of cases. A potential solution would be to make hepatitis E a systematically notifiable disease in Italy, as this would help get a better estimate of the national incidence and burden. Integrated surveillance needs to be systematised through the improvement of case finding and diagnostic capacity across all Italian regions. Importantly, the ‘One Health’ approach to integrated surveillance needs to also involve active surveillance of the animal sector (livestock and wildlife) and molecular surveillance also needs to be enhanced and standardised. This would allow us to characterise and possibly link strains isolated from humans, animals and environmental samples and interpret results in relation with epidemiological and clinical data.

## Notes

**Collaborating Group:** Luigina Ferrigno, Simonetta Crateri (Istituto Superiore di Sanità); Cristina Galli, Catia Tagliacarne (Università degli Studi di Milano); Maria Teresa Giordani, Erika Morelli, Anna Vit, Tosca Bertin, Serena Marinello, Giovanna De Checchi (Regione Veneto); Carla Zotti, Viola Amprino, Antonella Rossati (Regione Piemonte); Antonella Di Vito, Chiara Guidoni (Regione Toscana); Daniel Fiacchini, Katia Marinelli (Regione Marche); Mariarosaria Loffredo, Giovanna De Angelis (Regione Lazio); Annarita Citarella, Angelo Salomone Megnia (Regione Campania).
